# Distribution of serum uric acid concentration and its association with lipid profiles: a single-center retrospective study in children aged 3 to 12 years with adenoid and tonsillar hypertrophy

**DOI:** 10.1186/s12944-023-01806-2

**Published:** 2023-04-06

**Authors:** Jiating Yu, Xin Liu, Honglei Ji, Yawei Zhang, Hanqiang Zhan, Ziyin Zhang, Jianguo Wen, Zhimin Wang

**Affiliations:** 1 Henan Joint International Pediatric Urodynamic Laboratory, The First Affiliated Hospital of Zhengzhou University, Zhengzhou, Henan China; 2 Department of Clinical Laboratory, Key Clinical Laboratory of Henan Province, The First Affiliated Hospital of Zhengzhou University, Zhengzhou, Henan China; 3 NHC Key Laboratory of Reproduction Regulation, Shanghai Institute for Biomedical and Pharmaceutical Technologies, Shanghai, China; 4 Department of Endocrinology and Metabolic Diseases, The First Affiliated Hospital of Zhengzhou University, Zhengzhou, Henan China; 5 Department of Medical Record Management, The First Affiliated Hospital of Zhengzhou University, Zhengzhou, Henan China; 6 Department of Information, The First Affiliated Hospital of Zhengzhou University, Zhengzhou, Henan China

**Keywords:** Serum uric acid, Children, Lipid profiles, Dyslipidemia, Adenoid and tonsillar hypertrophy

## Abstract

**Background:**

Presently, there is no consensus regarding the optimal serum uric acid (SUA) concentration for pediatric patients. Adenoid and tonsillar hypertrophy is considered to be closely associated with pediatric metabolic syndrome and cardiovascular risk and is a common condition in children admitted to the hospital. Therefore, we aimed to evaluate the relationship between SUA and dyslipidemia and propose a reference range for SUA concentration that is associated with a healthy lipid profile in hospitalized children with adenoid and tonsillar hypertrophy.

**Methods:**

Preoperative data from 4922 children admitted for elective adenoidectomy and/or tonsillectomy surgery due to adenoid and tonsillar hypertrophy were collected. SUA concentrations were scaled to standard deviation (SD), and SUA deviations were expressed as SD from the mean SUA of children without dyslipidemia.

**Results:**

The mean SUA concentration of the participants was 4.27 ± 1.01 mg/dL, and the prevalence of hyperuricemia was 1.6% when it was defined using an SUA of ≥ 7.0 mg/dL. Participants with dyslipidemia (856, 17.4%) had a higher prevalence of hyperuricemia (3.4% vs. 1.2%, *P* < 0.001) and higher SUA concentrations (4.51 ± 1.15 vs. 4.22 ± 0.97 mg/dL, *P* < 0.001) than those with ortholiposis. The circulating lipid status of participants with SUAs < 1 SD below the mean value for the participants with ortholiposis (range 1.80–3.28 mg/dL) was more normal. Each 1-SD increase in SUA was associated with a 27% increase in the risk of dyslipidemia (OR = 1.270, 95% CI, 1.185–1.361). Adjustment for a number of potential confounders reduced the strength of the relationship, but this remained significant (OR = 1.125, 95% CI, 1.042–1.215). The higher risk of dyslipidemia was maintained for participants with SUAs > 1 SD above the mean value of the participants with ortholiposis.

**Conclusions:**

SUA was independently associated with dyslipidemia in children with adenoid and tonsillar hypertrophy, and an SUA < 1 SD below the mean value for patients with ortholiposis was associated with a healthy lipid profile.

**Supplementary Information:**

The online version contains supplementary material available at 10.1186/s12944-023-01806-2.

## Background

The worldwide epidemic of obesity is associated with a dysregulation of carbohydrate and lipid metabolism, which also promotes purine catabolism, leading to an accumulation of uric acid (UA) as an end-product [1, 2]. The prevalence of hyperuricemia has increased rapidly around the world in recent decades [3]. Many studies have demonstrated asymptomatic hyperuricemia or a high level of serum UA (SUA) as a risk factor for lifestyle-related diseases and all-cause mortality, demonstrating their close relationships with metabolic syndrome (MetS) and cardiovascular disease [4, 5]. The SUAs of some adolescents have been shown to be unexpectedly high, and the related chronic noncommunicable diseases are now developing at a younger age than previously [6–9]. Therefore, the prevention of high SUA concentrations and the minimization of metabolic risk in childhood may be beneficial. However, there is no consensus regarding the optimal SUA concentration in pediatric patients. SUA increases gradually with age, and it has been reported that the normal SUA of children is 1.0–1.5 mg/dL (60–90 μmol/L) lower than that of adults, but there is no validated reference range for the pediatric population [10].

Evaluating the metabolic value of SUA based on established metabolic risk indicators available from routine and standard laboratory tests should be considered alert values to correct. Dyslipidemia in childhood is a major component of pediatric MetS and is associated with early atherosclerotic phenotypes in adults [11]. The tonsils and adenoids act as the first line of defense of the immune system against swallowed or inhaled foreign pathogens, and their hypertrophy is commonly and closely associated with MetS and cardiovascular risk in children [12, 13]. Previous studies have focused on postoperative metabolic inertia in children with adenoidectomy, especially those with obesity [14]. According to the medical record system of our hospital, elective adenoidectomy owing to adenoid and tonsillar hypertrophy was the most common admittance condition in children. Knowledge of the relationship between SUA and the circulating lipid status of children with adenoid and tonsillar hypertrophy might improve strategies to prevent pediatric hyperuricemia.

We performed a study in children with adenoid and tonsillar hypertrophy with the aims of (1) identifying a suitable reference value of SUA that is associated with a healthy lipid profile in children and (2) evaluating the relationship between SUA and dyslipidemia to identify the SUA concentrations associated with metabolic alerts in children.

## Methods

### Study population

We retrospectively reviewed the electronic medical records of children aged 3–12 years who were admitted for elective adenoidectomy and/or tonsillectomy surgery in the Otorhinolaryngologic Department at the First Affiliated Hospital of Zhengzhou University. A data set from the first page of medical records between January 2018 and August 2022 was collected, and cases were identified using International Classification of Diseases, 10th Revision, Current Procedural Terminology, and Healthcare Common Procedure Coding System codes. A total of 8583 patients were admitted, among whom 4922 participants (3174 boys and 1748 girls) were finally enrolled according to the following inclusion and exclusion criteria (Supplementary Fig. 1). Institutional review board approval was obtained, and consent requirements were waived. Personal information was anonymized during data collection and analysis.

### Inclusion criteria

The inclusion criteria were (1) aged 3–12 years; (2) initial hospitalization at the First Affiliated Hospital of Zhengzhou University; (3) results of routine preoperative testing available, including complete blood count, routine urinalysis data, electrolytes, liver enzymes (gamma-glutamyl transpeptidase, GGT; alkaline phosphatase, ALP; albumin; prealbumin) and kidney function (creatinine, Cr; urea) within the sex- and age-specific normal ranges [15, 16]; (4) thyroid function (free triiodothyronine, free thyroxin and thyroid stimulating hormone) within the sex- and age-specific normal ranges [17]; and (5) adenoidectomy performed because of adenoid and tonsillar hypertrophy.

### Exclusion criteria

The exclusion criteria were (1) incomplete electronic records, including laboratory data; (2) a history of arthritis or arthralgia, including gout; (3) endocrine disorders or syndromes affecting metabolic status, including disorders of the endocrine pancreas, pituitary, adrenal glands, thyroid, and parathyroid; (4) a history of regular medical visits or routine medication; (5) adenoidectomy because of recurrent ear infections or otitis; and (6) a history of previous adenoidectomy, revision surgery, or other major surgical procedure.

### Data collection

Demographic data (age, sex, weight and height) were collected. The body mass index (BMI) z score was estimated with the World Health Organization (WHO) child growth standards (2–5 years old) and WHO child growth reference (5–19 years old) and calculated on the website Age-based Pediatric Growth Reference Charts [18]. Preoperative clinical laboratory tests were routinely performed after an overnight fast, and the following data were collected: SUA, fasting plasma glucose (FPG), total cholesterol (TC), triglyceride (TG), high-density lipoprotein cholesterol (HDL-C), low-density lipoprotein cholesterol (LDL-C), alanine aminotransferase (ALT), aspartate aminotransferase (AST), GGT, ALP, urea and Cr. They were measured using an automatic biochemistry analyzer (Roche, Germany) with standard methods. Non-HDL-C was calculated by subtracting HDL-C from TC.

### Definition of dyslipidemia and categorization of SUA

Dyslipidemia was defined using one or a combination of the following: (1) TC ≥ 5.18 mmol/L (200 mg/dL, hypercholesterolemia); (2) TG ≥ 1.47 mmol/L (130 mg/dL, hypertriglyceridemia); (3) HDL-C < 1.03 mmol/L (40 mg/dL, low HDL-C); (4) LDL-C ≥ 4.1 mmol/L (160 mg/dL, high LDL-C); and (5) non-HDL-C ≥ 3.76 mmol/L (145 mg/dL, high non-HDL-C) [19]. Participants with one or more of these abnormalities were placed in a Dyslipidemia group, and the others were placed into an Ortholiposis group.

We calculated the mean and standard deviation (SD) of the SUA concentration for the Ortholiposis group and then scaled continuous SUA levels to this SD. We then categorized the participants using the number of SDs by which their SUA concentrations differed from the mean SUA concentration of the Ortholiposis group.

### Statistical analyzes

Statistical analyzes were performed using SPSS 25.0 for Windows (SPSS Inc., Chicago, IL). Normally distributed continuous variables are presented as the mean ± SD, and categorical variables are presented as proportions. Significant differences between two groups were tested by Student’s t test, Mann‒Whitney U test or Pearson chi-square test. Significant differences between multiple groups were compared using the chi-square test for trend, one-way analysis of variance or Welch's t test, and multiple comparisons between groups were analyzed with Bonferroni correction. Pearson correlation analysis was used to analyze the correlation between continuous variables. Univariate binary logistic regression analysis was used to assess odds ratios (ORs) and corresponding 95% confidence intervals (CIs) of dyslipidemia. Unadjusted and multivariate adjusted binary logistic regression analyzes were used to investigate the association between SUA deviations and dyslipidemia with all potential independent confounders determined by univariate analysis. The statistical tests were two tailed, and a *P* value < 0.05 was regarded as statistically significant.

## Results

### Characteristics of the participants

**Fig. 1:**
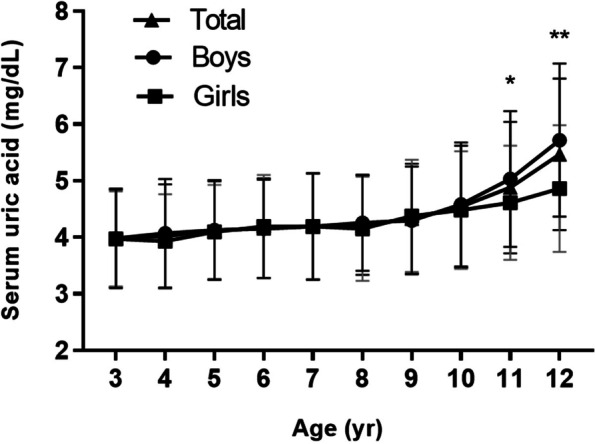
Change in the mean and SD of SUA with age by sex. Sex differences in SUA concentrations at the same age: *P < 0.05, **P < 0.001

Of the 4922 patients enrolled, 856 (17.4%) were placed into the Dyslipidemia group (Table 1). The SUA concentration ranged from 1.80 to 9.19 mg/dL, increased gradually between 3 and 10 years of age, and then increased markedly between 11 and 12 years of age (Fig. 1; Supplementary Table 1). There were no sex differences, other than in 11- and 12-year-olds, among whom the SUAs of boys were significantly higher than those of girls (Fig. 1). For the entire cohort, the mean SUA was 4.27 ± 1.01 mg/dL, the mean TC was 3.85 ± 0.67 mmol/L, the mean TG was 0.87 ± 0.52 mmol/L, the mean HDL-C was 1.40 ± 0.30 mmol/L, the mean LDL-C was 2.16 ± 0.57 mmol/L, the mean non-HDL-C was 2.45 ± 0.62 mmol/L and the mean FPG was 4.44 ± 0.47 mmol/L (Table 1). Pearson correlation analysis showed a significant negative correlation between the SUA and HDL-C concentrations and a positive correlation of SUA with the TG and non-HDL-C concentrations (Supplementary Table 2).

**Table 1:** Characteristics of the study subjects and comparison of characteristics between children in the Ortholiposis and Dyslipidemia

Characteristics	Total (*n* = 4922)	Ortholiposis (*n* = 4066)	Dyslipidemia (*n* = 856)	*P* value
Age, yr	6.7 ± 2.6	6.7 ± 2.5	7.0 ± 2.9	0.001
Boys [n (%)]	3174 (64.5)	2673 (65.7)	501 (58.5)	< 0.001
BMI z score	0.36 ± 1.17	0.32 ± 1.17	0.56 ± 1.13	< 0.001
SUA, mg/dL	4.27 ± 1.01	4.22 ± 0.97	4.51 ± 1.15	< 0.001
Hyperuricemia defined as ≥ 5.5 mg/dL (%)	542 (11.0)	390 (9.6)	152 (17.8)	< 0.001
Hyperuricemia defined as ≥ 7.0 mg/dL (%)	78 (1.6)	49 (1.2)	29 (3.4)	< 0.001
FPG, mmol/L	4.44 ± 0.47	4.44 ± 0.47	4.47 ± 0.48	0.091
TC, mmol/L	3.85 ± 0.67	3.81 ± 0.56	4.04 ± 1.02	< 0.001
TG, mmol/L	0.87 ± 0.52	0.75 ± 0.25	1.46 ± 0.91	< 0.001
HDL-C, mmol/L	1.40 ± 0.30	1.45 ± 0.26	1.14 ± 0.33	< 0.001
Non-HDL-C, mmol/L	2.45 ± 0.62	2.35 ± 0.51	2.90 ± 0.85	< 0.001
LDL-C, mmol/L	2.16 ± 0.57	2.11 ± 0.48	2.44 ± 0.83	< 0.001
ALT, U/L	13.04 ± 4.25	12.79 ± 4.02	14.24 ± 5.06	< 0.001
AST, U/L	23.54 ± 5.08	23.64 ± 4.96	23.06 ± 5.60	0.005
GGT, U/L	10.65 ± 3.68	10.36 ± 3.28	12.05 ± 4.94	< 0.001
ALP, U/L	241.41 ± 60.84	240.48 ± 59.74	245.82 ± 65.66	0.028
Serum urea, μmol/L	4.58 ± 0.97	4.59 ± 0.97	4.52 ± 0.99	0.058
Serum Cr, μmol/L	38.21 ± 8.02	38.17 ± 7.86	38.39 ± 8.74	0.488

For the participants with dyslipidemia, the mean SUA was 4.51 ± 1.15 mg/dL, the mean TC was 4.04 ± 1.02 mmol/L, the mean TG was 1.46 ± 0.91 mmol/L, the mean HDL-C was 1.14 ± 0.33 mmol/L, the mean LDL-C was 2.44 ± 0.83 mmol/L, the mean non-HDL-C was 2.90 ± 0.85 mmol/L and the mean FPG was 4.47 ± 0.48 mmol/L (Table 1). Compared with participants in the Ortholiposis group, those with dyslipidemia were older; had a significantly higher prevalence of hyperuricemia; and had higher BMI z score, SUA, TC, TG, LDL-C, non-HDL-C, ALT, GGT and ALP. HDL-C and AST were significantly lower in the Dyslipidemia group (Table 1).

### SUA deviations and their relative clinical characteristics

As shown in Table 1, the mean and SD of SUA in participants with ortholiposis were 4.22 and 0.97 mg/dL, respectively. The spread in the SUA data for all 4922 enrolled participants ranged from -2.5 SD to + 5.3 SD. Accordingly, the participants were placed into five groups: Group 1, defined as SUA < 1 SD below the mean value for the Ortholiposis group (from -2.5 SD to -1 SD); Group 2, with SUAs between 1 SD below the mean value for the Ortholiposis group and the mean (from -1 SD to 0 SD); Group 3, with SUAs between the mean value for the Ortholiposis group and 1 SD above the mean (from 0 SD to + 1 SD); Group 4, with SUAs between 1 and 2 SDs above the mean for the Ortholiposis group (from + 1 SD to + 2 SD); and Group 5, with SUAs > 2 SD above the mean for the Ortholiposis group ( from + 2 SD to + 5.3 SD). In Group 1, the SUA concentrations ranged from 1.80 to 3.28 mg/dL, with a mean value of 2.92 ± 0.30 mg/dL; in Group 2, the SUA ranged from 3.29 to 4.22 mg/dL, with a mean value of 3.78 ± 0.27 mg/dL; in Group 3, the SUA ranged from 4.23 to 5.16 mg/dL, with a mean value of 4.65 ± 0.26 mg/dL; in Group 4, the SUA ranged from 5.17 to 6.10 mg/dL, with a mean value of 5.54 ± 0.26 mg/dL; and in Group 5, the SUA ranged from 6.12 to 9.19 mg/dL, with a mean value of 6.84 ± 0.70 mg/dL.

**Fig. 2:**
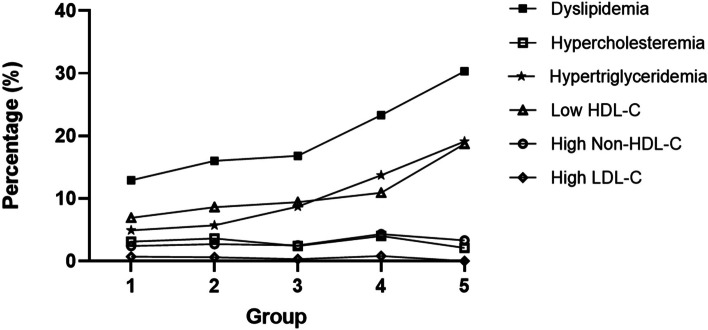
Percentage of dyslipidemia and abnormal lipid profiles among groups with SUA deviations

Among SUA deviation groups, significant differences were observed in terms of age, sex, BMI z score, FPG, IFG, TG, HDL-C, non-HDL-C, ALT, AST, GGT, ALP, urea and Cr (Table 2). The prevalence of dyslipidemia increased with group number, particularly the prevalence of hypertriglyceridemia and low HDL-C (Fig. 2). Groups 1 and 2 were younger; had significantly higher HDL-C; lower BMI z score, TG, urea and Cr; and a lower prevalence of hypertriglyceridemia than Groups 3, 4, and 5. In addition, Group 1 was younger; had significantly lower BMI z scores, ALT, GGT, ALP, Cr, FPG, TC, LDL-C and non-HDL-C; and a lower prevalence of dyslipidemia than Group 2. Therefore, Group 1 (SUA deviations between -2.5 and -1 SD) was defined as the most suitable reference group, and the other groups were compared with this group in further analyzes.

**Table 2:** Comparison of characteristics between children in different SUA deviation groups

Characteristics	SUA deviation groups					*P* value
	Group 1 < -1SD (*n* = 742)	Group 2 -1 ≥ SD < 0 (*n* = 1902)	Group 3 0 ≥ SD < 1 (*n* = 1431)	Group 4 1 ≥ SD < 2 (*n* = 606)	Group 5 ≥ 2SD (*n* = 241)	
Age, yr	6.0 ± 2.3^bcde^	6.3 ± 2.3^acde^	6.9 ± 2.6^abde^	7.6 ± 2.7^abce^	9.1 ± 2.9^abcd^	< 0.001
Boys [n (%)]	453 (61.1)	1229 (64.6)	905 (63.2)	409 (67.5)	178 (73.9)	0.001
BMI z score	0.09 ± 1.27^bcde^	0.26 ± 1.19^acde^	0.46 ± 1.11^abde^	0.62 ± 1.05^abc^	0.82 ± 0.92^abc^	< 0.001
SUA, mg/dL	2.92 ± 0.30^bcde^	3.78 ± 0.27^acde^	4.65 ± 0.26^abde^	5.54 ± 0.26^abce^	6.84 ± 0.70^abcd^	< 0.001
FPG, mmol/L	4.40 ± 0.41^ce^	4.43 ± 0.47^e^	4.46 ± 0.51^a^	4.44 ± 0.46^e^	4.54 ± 0.49^abd^	< 0.001
TC, mmol/L	3.83 ± 0.69	3.85 ± 0.69	3.85 ± 0.62	3.88 ± 0.67	3.78 ± 0.69	0.421
TG, mmol/L	0.80 ± 0.48^cde^	0.82 ± 0.41^cde^	0.88 ± 0.57^abde^	0.99 ± 0.59^abce^	1.16 ± 0.71^abcd^	< 0.001
HDL-C, mmol/L	1.44 ± 0.31^cde^	1.42 ± 0.31^cde^	1.39 ± 0.28^abe^	1.37 ± 0.29^abe^	1.25 ± 0.26^abcd^	< 0.001
Non-HDL-C, mmol/L	2.39 ± 0.62^de^	2.43 ± 0.63^d^	2.46 ± 0.58	2.51 ± 0.64^ab^	2.53 ± 0.64^a^	0.001
LDL-C, mmol/L	2.13 ± 0.59	2.16 ± 0.59	2.17 ± 0.54	2.19 ± 0.57	2.15 ± 0.56	0.313
ALT, U/L	12.06 ± 3.39^bcde^	12.67 ± 3.95^acde^	13.23 ± 4.38^abde^	14.09 ± 4.80^abce^	15.23 ± 5.26^abcd^	< 0.001
AST, U/L	24.18 ± 5.26^cde^	24.11 ± 5.07^cde^	23.24 ± 4.78^abde^	22.57 ± 5.24^abce^	21.31 ± 4.92^abcd^	< 0.001
GGT, U/L	9.30 ± 2.56^bcde^	10.02 ± 2.95^acde^	11.00 ± 3.75^abde^	12.20 ± 4.37^abce^	13.82 ± 5.50^abcd^	< 0.001
ALP, U/L	227.75 ± 55.15^bcde^	235.49 ± 53.92^acde^	242.69 ± 58.55^abde^	258.21 ± 69.09^abce^	280.37 ± 88.92^abcd^	< 0.001
Serum urea, μmol/L	4.46 ± 0.95^cde^	4.52 ± 0.98^cde^	4.63 ± 0.97^ab^	4.72 ± 0.95^ab^	4.76 ± 0.98^ab^	< 0.001
Serum Cr, μmol/L	35.03 ± 7.07^bcde^	36.91 ± 7.24^acde^	39.08 ± 7.77^abde^	41.04 ± 8.11^abce^	45.89 ± 9.82^abcd^	< 0.001
Dyslipidemia [n (%)]	96 (12.9)	305 (16.0)	241 (16.8)	141 (23.3)	73 (30.3)	< 0.001
Hypercholesteremia [n (%)]	23 (3.1)	69 (3.6)	34 (2.4)	24 (4.0)	5 (2.1)	0.500
Hypertriglyceridemia [n (%)]	36 (4.9)	109 (5.7)	125 (8.7)	83 (13.7)	46 (19.1)	< 0.001
Low HDL-C [n (%)]	51 (6.9)	163 (8.6)	134 (9.4)	66 (10.9)	45 (18.7)	< 0.001
High Non-HDL-C [n (%)]	18 (2.4)	52 (2.7)	36 (2.5)	26 (4.3)	8 (3.3)	0.112
High LDL-C [n (%)]	5 (0.7)	11 (0.6)	5 (0.3)	5 (0.8)	0 (0.0)	0.467

Compared with Group 1, ^a^ *P* < 0.05; compared with Group 2, ^b^ *P* < 0.05; compared with Group 3, ^c^ *P* < 0.05; compared with Group 4, ^d^ *P* < 0.05; compared with Group 5, ^e^ *P* < 0.05 (Bonferroni correction)

### Relationship between SUA and dyslipidemia

Univariate logistic regression analysis showed that the following variables were risk factors for dyslipidemia: age, sex, BMI z score, SUA, ALT, AST, GGT and ALP (Supplementary Table 3). Unadjusted binary logistic regression analysis showed that each additional 1-SD increase in SUA was associated with a 27% increase in the risk of dyslipidemia (Table 3, Model 1: OR = 1.270, 95% CI, 1.185–1.361). As shown in Table 3, compared with the SUA deviations in Group 1, those in Groups 2, 3, 4 and 5 were significantly associated with higher risks of dyslipidemia (Group 2: OR, 1.285, 95% CI, 1.004–1.645; Group 3: OR, 1.363, 95% CI, 1.056–1.759; Group 4: OR, 2.040, 95% CI, 1.534–2.714; Group 5: OR, 2.924, 95% CI, 2.064–4.143). Adjustment of the data for age and sex did not alter the association between the deviation from the mean SUA of the Ortholiposis group and the risk of dyslipidemia (Table 3, Model 2: OR = 1.259, 95% CI, 1.171–1.353). After further adjustment for BMI z score, a significantly higher risk remained for Groups 4 and 5 (Table 3, Model 3: Group 4, OR = 1.841, 95% CI, 1.373–2.470; Group 5, OR = 2.541, 95% CI, 1.762–3.665) but disappeared for Groups 2 and 3. Finally, after further adjustment for ALT, AST, GGT and ALP, the strength of this association was weakened, but it remained statistically significant (Table 3, Model 4: OR = 1.125, 95% CI, 1.042–1.215; Group 4, OR = 1.475, 95% CI, 1.090–1.996; Group 5, OR = 1.870, 95% CI, 1.276–2.740).

**Table 3:** Association between SUA deviations and dyslipidemia

	Model 1		Model 2		Model 3		Model 4	
	OR (95%CI)	*P* value	OR (95%CI)	*P* value	OR (95%CI)	*P* value	OR (95%CI)	*P* value
SUA deviations	1.270 (1.185–1.361)	< 0.001	1.259 (1.171–1.353)	< 0.001	1.220 (1.134–1.314)	< 0.001	1.125 (1.042–1.215)	0.003
Group 1 (< -1SD)	1.00	―	1.00	―	1.00	―	1.00	―
Group 2 (-1 ≥ SD < 0)	1.285 (1.004–1.645)	0.046	1.292 (1.009–1.656)	0.042	1.260 (0.983–1.615)	0.068	1.202 (0.935–1.544)	0.151
Group 3 (0 ≥ SD < 1)	1.363 (1.056–1.759)	0.017	1.345 (1.040–1.739)	0.024	1.263 (0.974–1.636)	0.078	1.100 (0.845–1.432)	0.480
Group 4 (1 ≥ SD < 2)	2.040 (1.534–2.714)	< 0.001	2.014 (1.506–2.693)	< 0.001	1.841 (1.373–2.470)	< 0.001	1.475 (1.090–1.996)	0.012
Group 5 (≥ 2SD)	2.924 (2.064–4.143)	< 0.001	2.861 (1.992–4.109)	< 0.001	2.541 (1.762–3.665)	< 0.001	1.870 (1.276–2.740)	0.001

Model 1 was unadjusted; model 2 was adjusted for age and sex; model 3 was adjusted for BMI z score as well as age and sex; and model 4 was further adjusted for ALT, AST, GGT, ALP and variables in model 3

## Discussion

In the present study of children aged 3–12 years old with adenoid and tonsillar hypertrophy, we found that their mean SUA concentration was 4.27 ± 1.01 mg/dL and that there was no difference between the sexes, except in 11- and 12-year-olds, in which the SUAs of boys were significantly higher than those of girls. We also recommended a reference range of SUA that was associated with a healthy lipid profile according to our study of the deviations from the mean of individuals with ortholiposis. We also showed that SUA was an independent risk factor for dyslipidemia in children with adenoid and tonsillar hypertrophy. Notably, after adjusting for a number of other potential confounders, the positive association between SUA and dyslipidemia remained.

SUA was found to increase markedly around puberty, and within the following adolescent years, it likely reached adult concentrations [6]. Hyperuricemia in adults is defined using an SUA concentration > 7 mg/dL (420 μmol/L) in men and 6 mg/dL (360 μmol/L) in women, irrespective of the presence or absence of symptoms or signs of the deposition of urate crystals [1, 20]. In one cross-sectional survey performed in 2017–2018 in China, hyperuricemia was defined using an SUA concentration > 5.5 mg/dL in Chinese adolescents aged 13–19 years [8]. In addition, a large pooled cross-sectional study generated an estimated overall prevalence of hyperuricemia of 23.3% (26.6% in boys and 19.8% in girls) in Chinese children and adolescents aged 3–19 years during 2009–2019, using the adult reference range [6]. Ito et al. reported that the prevalence of gout and asymptomatic hyperuricemia was 0.04% among children aged 0–18 years in Japan on the basis of a survey of the 2016–2017 health insurance claims database and a definition of hyperuricemia using an SUA > 7 mg/dL [7]. In the present study, the prevalence of hyperuricemia was 11.2% when hyperuricemia was defined using an SUA > 5.5 mg/dL [8] and 1.6% when it was defined using an SUA ≥ 7 mg/dL.

Although complications of hyperuricemia are extremely rare in children, the metabolic status of pediatric patients is recognized as important for long-term outcomes [11]. Currently, there is no published reference range for SUA in pediatric patients. Li et al. reported that the mean SUA concentration of children aged 3–6 years in China is 4.08 ± 0.89 mg/dL and that there are sex differences (4.15 ± 0.90 mg/dL for boys and 4.00 ± 0.88 mg/dL for girls) [21], whereas Lee et al. reported a mean SUA of 4.2 ± 0.8 mg/dL, without sex differences, in 6-year-old children in Korea [22]. The present findings supported that sex differences in SUA concentration emerge around puberty.

Dyslipidemia is associated with worse cardiovascular outcomes [23]. Lipid-lowering therapy with statin and nonstatin agents, including proprotein convertase subtilisin/kexin 9 inhibitors (PCSK9i), correlates linearly with a decreased risk of cardiovascular events [24]. The relationship between SUA and lipid profiles was revealed. Russo et al. showed a close relationship between SUA and TG in healthy individuals [25]. Children with higher TG levels (≥ 1.70 mmol/L) had a higher prevalence of hyperuricemia [21]. Both the SUA and TC concentrations were found to increase with age. SUA increased gradually between 3 and 11 years of age and then increased markedly at approximately 11–15 years of age until it reached an adult concentration [6]. The TC concentrations increase from birth, stabilize at approximately 2 years old, and reach a peak prior to puberty [26]. In the present study, we found significant relationships between SUA and lipid concentrations in children with adenoid and tonsillar hypertrophy; specifically, we found positive correlations of SUA with TG and non-HDL-C and a negative correlation between SUA and HDL-C. Lipoprotein concentrations were not measured routinely preoperatively and therefore could not be included in the present retrospective study. Nevertheless, we have shown significant correlations between SUA and lipid concentrations related to ApoE, including TG and HDL-C [27, 28].

Adenoids and tonsils are the first line of defense against pathogenic agents and participate in the immune system. Studies have found that both local and systemic inflammation are strongly associated with the morbidity of adenoid and tonsillar hypertrophy in children. Compared to healthy children, children with chronic adenoid and tonsillar hypertrophy were under significant oxidative stress and had significantly increased levels of proinflammatory cytokines [29, 30]. Chronic and recurrent adenoid and tonsillar hypertrophy could cause harmful effects on untreated children, including upper airway obstruction, chronic intermittent hypoxia and sleep-disordered breathing [31]. As a marker of hypoxic stress, the UA concentration is associated with inflammatory stimulation [32, 33]. Hypoxia stress affects glycolysis and gluconeogenesis with the accumulation of pyruvate and the release of purine intermediates (adenosine, inosine, hypoxanthine, and xanthine), leading to the overproduction of UA, the final metabolite of purine catabolism [32].

We also found that high BMI z scores were associated with high SUA concentrations. The relationship between SUA and adipose tissue has been well established [34, 35]. Chronic high SUA concentrations cause a pro-inflammatory state in adipose tissue by inducing the release of monocyte chemoattractant protein 1, inducing a vicious cycle [33]. In addition, studies have shown that adipose tissue produces UA [36]. The activity of the enzyme xanthine oxidoreductase, which converts xanthine and hypoxanthine to UA, is high during obesity [33, 36]. Compared to normal-weight children, obese children have higher SUA concentrations [21]. In a cross-sectional study, Higgins et al. showed a link between excess adiposity and the circulating concentrations of routinely assessed lipid species and SUA in healthy children [37]. In the present study, we found that both SUA and BMI z score were independent risk factors for dyslipidemia in children with adenoid and tonsillar hypertrophy. After correcting for BMI z score (Table 3, model 3 and model 4), the higher risk of dyslipidemia remained in participants with SUAs > 1 SD above the mean value for participants with ortholiposis but not in those with SUAs < 1 SD above the mean. In addition, and similar to the findings of previous studies [38], we found strong associations between SUA and liver enzymes (Supplement Table 3). Furthermore, after adjustment for liver enzyme activities (Table 3, model 4), the association between SUA and dyslipidemia remained significant, although it was weaker. Collectively, this study indicated the impact of childhood obesity and liver enzymes on SUA and its impact on dyslipidemia risk. Further prospective studies are needed to determine whether the SUA concentration and the relationships with liver enzymes in individuals with obesity reflect or might predict postoperative metabolic inertia in children undergoing adenotonsillectomy.

### Study strengths and limitations

To date, limited evidence has been provided regarding appropriate childhood screening strategies and follow-up interventions targeting SUA to reduce the incidence of adult gout and delay the onset of related metabolic events. Although the children admitted for elective adenoidectomy who were recruited for the present study might not be representative of the wider affected or at-risk population, to our knowledge, our study is the first to provide information on SUA levels and their metabolic effect on adenoid and tonsillar hypertrophy, which is a common condition with high metabolic risks in pediatrics and is the most common condition for pediatric hospitalization. Some limitations to the present retrospective study from a single institution from a specific region in China should be acknowledged. First, selection and recall biases are inherent to retrospective studies, and it was difficult to obtain accurate information from the medical records of the participants. In addition, the medical records contained limited information regarding the assessment of growth and development status and the family history of genetic conditions. The relationship between SUA and dyslipidemia in some of the participants might have been confounded by the effects of inherited variants in metabolic genes. Second, there was a lack of detailed information regarding lipoprotein and urate excretion, including the urate/Cr clearance ratio. Third, as a Chinese single-center study, the findings might not be generalizable to different populations and races, particularly those including children with different age distributions. Fourth, we were unable to extrapolate our findings to children in the community, as the data were collected from a medical institution in China. Finally, the relationships between UA and other MetS components were not evaluated in the present study. Future research is essential to determine the effects of SUA concentrations on other MetS components in children with adenoid and tonsillar hypertrophy. In the future, a larger multicenter study is needed to confirm these findings, and a prospective cohort study will be required to implement appropriate interventions.

## Conclusion

SUA concentration was independently associated with dyslipidemia in children with adenoid and tonsillar hypertrophy, and SUA less than 1 SD below the mean SUA of children with ortholiposis was associated with a healthy lipid profile. As one most common admittance condition, pediatric adenoid and tonsillar hypertrophy is closely associated with metabolic syndrome and cardiovascular risk. Our study proposed a reference SUA range associated with a healthy lipid profile in hospitalized children with adenoid and tonsillar hypertrophy, and provided support for appropriate screening and management strategies targeting SUA associated with metabolic alerts in children.

## Supplementary Information

**Additional file 1:**  **Supplementary Figure 1**. A flow chart of this study.

**Additional file 2: Supplementary Table 1.** Comparison of SUA concentrations (mg/dL) with sex by age. Compared with 3 yr, ^a^*P*<0.05; compared with 4 yr, ^b^*P*<0.05; compared with 5 yr, ^c^*P*<0.05; compared with 6 yr, ^d^*P*<0.05; compared with 7 yr, ^e^*P*<0.05; compared with 8 yr, ^f^*P*<0.05; compared with 9 yr, ^g^*P*<0.05; compared with 10 yr, ^h^*P*<0.05; compared with 11 yr, ^i^*P*<0.05; compared with 12 yr, ^j^*P*<0.05 (Bonferroni correction). **Supplementary Table 2.** Correlation analysis between SUA concentrations and other continuous variables. **Supplementary Table 3.** Univariate regression analysis for dyslipidemia.

## Data Availability

The datasets used and/or analyzed during the current study are available from the corresponding authors on reasonable request.

## Abbreviations

ALP: Alkaline phosphatase
ALT: Alanine aminotransferase
AST: Aspartate aminotransferase
BMI: Body mass index
CI: Confidence interval
Cr: Creatinine
FPG: Fasting plasma samples
GGT: Gamma-glutamyl transpeptidase
HDL-C: High-density lipoprotein cholesterol
LDL-C: Low-density lipoprotein cholesterol
MetS: Metabolic syndrome
OR: Odds ratio
SD: Standard deviation
SUA: Serum uric acid
TC: Total cholesterol
TG: Triglyceride
UA: Uric acid
