# Supplementary figures and images for: Distribution of serum uric acid concentration and its association with lipid profiles: a single-center retrospective study in children aged 3 to 12 years with adenoid and tonsillar hypertrophy
Source: Lipids Health Dis. 2023 Apr 6;22:48. doi: 10.1186/s12944-023-01806-2 (PMC10077755; doi:10.1186/s12944-023-01806-2)

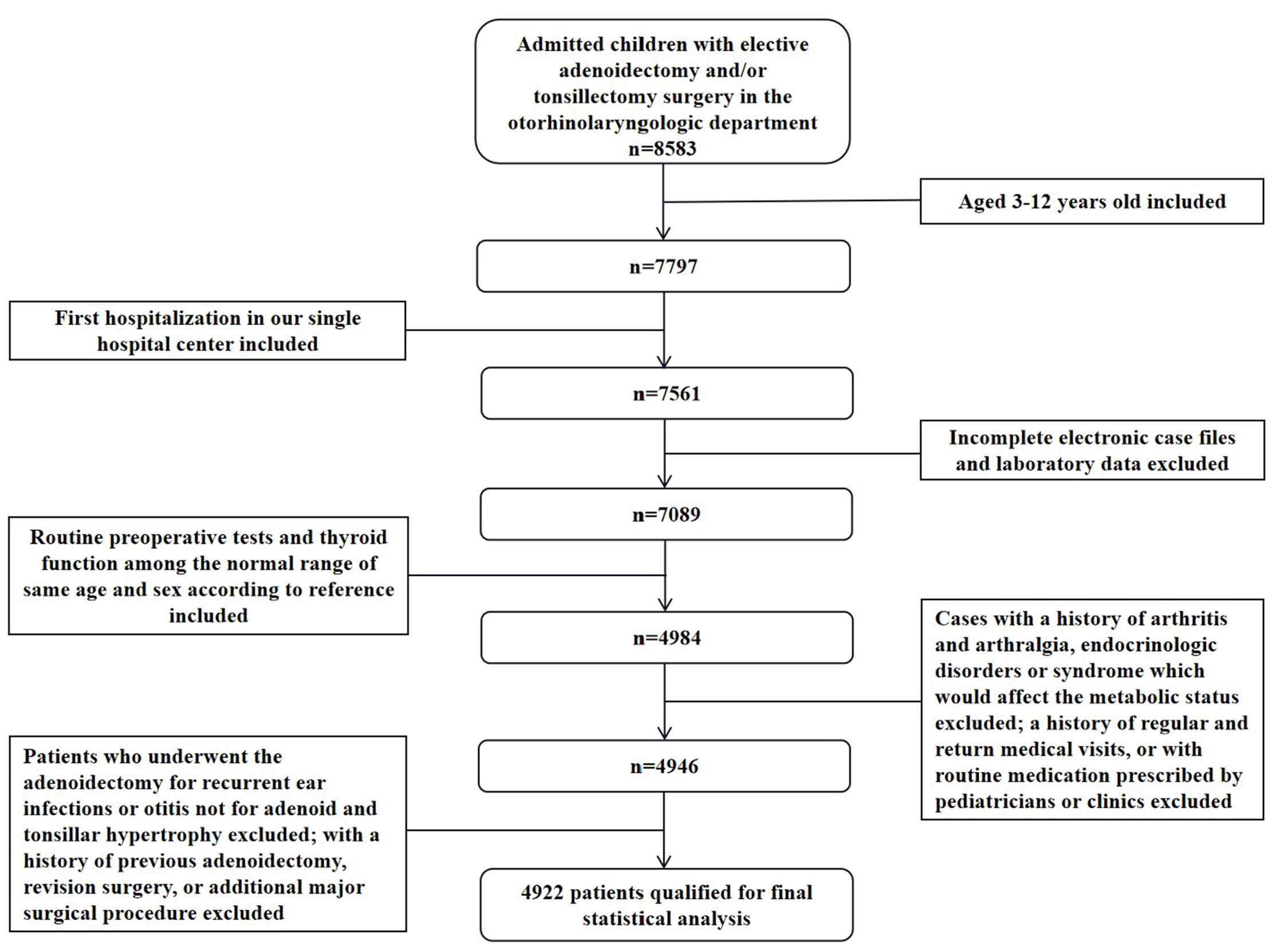

Supplement: Supplementary file 1 — Additional file 1: Supplementary Figure 1. A flow chart of this study. [file 12944_2023_1806_MOESM1_ESM.jpg]
