# Supplementary material for: Distribution of serum uric acid concentration and its association with lipid profiles: a single-center retrospective study in children aged 3 to 12 years with adenoid and tonsillar hypertrophy
Source: Lipids Health Dis. 2023 Apr 6;22:48. doi: 10.1186/s12944-023-01806-2 (PMC10077755; doi:10.1186/s12944-023-01806-2)
Supplement: Supplementary file 2 — Additional file 2: Supplementary Table 1. Comparison of SUA concentrations (mg/dL) with sex by age. Compared with 3 yr, aP<0.05; compared with 4 yr, bP<0.05; compared with 5 yr, cP<0.05; compared with 6 yr, dP<0.05; compared with 7 yr, eP<0.05; compared with 8 yr, fP<0.05; compared with 9 yr, gP<0.05; compared with 10 yr, hP<0.05; compared with 11 yr, iP<0.05; compared with 12 yr, jP<0.05 (Bonferroni correction). Supplementary Table 2. Correlation analysis between SUA concentrations and other continuous variables. Supplementary Table 3. Univariate regression analysis for dyslipidemia. [file 12944_2023_1806_MOESM2_ESM.doc]

| **Supplementary Table 1.** Comparison of SUA concentrations (mg/dL) with sex by age. | | | | | |
| --- | --- | --- | --- | --- | --- |
| Age (yr) | n | Total  (n=4922) | Girl  (n=3174) | Boy  (n=1748) | *P* value |
| 3 | 512 | 3.98±0.86^efghij^ | 3.97±0.84 | 3.98±0.88 | 0.822 |
| 4 | 673 | 4.02±0.92^fghij^ | 3.93±0.83 | 4.07±0.96 | 0.050 |
| 5 | 710 | 4.12±0.87^ghij^ | 4.10±0.83 | 4.13±0.88 | 0.757 |
| 6 | 593 | 4.16±0.88^hij^ | 4.19±0.91 | 4.15±0.87 | 0.593 |
| 7 | 630 | 4.19±0.94^ahij^ | 4.19±0.93 | 4.19±0.94 | 0.985 |
| 8 | 528 | 4.22±0.88^abhij^ | 4.15±0.92 | 4.26±0.85 | 0.158 |
| 9 | 422 | 4.33±0.97^abcij^ | 4.38±0.99 | 4.30±0.95 | 0.450 |
| 10 | 332 | 4.54±1.08^abcdefhij^ | 4.48±1.04 | 4.58±1.10 | 0.410 |
| 11 | 299 | 4.88±1.16^abcdefghij^ | 4.61±1.01 | 5.03±1.20 | 0.003 |
| 12 | 223 | 5.47±1.34^abcdefghij^ | 4.86±1.12 | 5.72±1.35 | ＜0.001 |

Compared with 3 yr, ^a^*P*<0.05; compared with 4 yr, ^b^*P*<0.05; compared with 5 yr, ^c^*P*<0.05; compared with 6 yr, ^d^*P*<0.05; compared with 7 yr, ^e^*P*<0.05; compared with 8 yr, ^f^*P*<0.05; compared with 9 yr, ^g^*P*<0.05; compared with 10 yr, ^h^*P*<0.05; compared with 11 yr, ^i^*P*<0.05; compared with 12 yr, ^j^*P*<0.05 (Bonferroni correction).

| **Supplementary Table 2.** Correlation analysis between SUA concentrations and other continuous variables. | | | |
| --- | --- | --- | --- |
| Variable | n | Pearson coefficient | *P* value |
| Age, yr | 4922 | 0.290 | <0.001 |
| BMI z-score | 4922 | 0.169 | <0.001 |
| FPG, mmol/L | 4922 | 0.054 | <0.001 |
| TC, mmol/L | 4922 | 0.001 | 0.921 |
| TG, mmol/L | 4922 | 0.173 | <0.001 |
| HDL-C, mmol/L | 4922 | -0.140 | <0.001 |
| Non-HDL-C, mmol/L | 4922 | 0.069 | <0.001 |
| LDL-C, mmol/L | 4922 | 0.027 | 0.058 |
| ALT, U/L | 4922 | 0.175 | <0.001 |
| AST, U/L | 4922 | -0.148 | <0.001 |
| GGT, U/L | 4922 | 0.317 | <0.001 |
| ALP, U/L | 4922 | 0.203 | <0.001 |
| Serum urea, μmol/L | 4922 | 0.090 | <0.001 |
| Serum Cr, μmol/L | 4922 | 0.328 | <0.001 |

| **Supplementary Table 3.** Univariate regression analysis for dyslipidemia. | | | | |
| --- | --- | --- | --- | --- |
| Characteristics | β | SE | OR (95%CI) | *P* value |
| SUA, mg/dL | 0.268 | 0.035 | 1.308 (1.220-1.402) | <0.001 |
| Age, yr | 0.052 | 0.014 | 1.053 (1.024-1.083) | <0.001 |
| Sex | -0.307 | 0.077 | 0.735 (0.633-0.855) | <0.001 |
| BMI z-score | 0.187 | 0.035 | 1.206 (1.126-1.291) | <0.001 |
| FPG, mmol/L | 0.133 | 0.078 | 1.142 (0.979-1.331) | 0.091 |
| ALT, U/L | 0.071 | 0.008 | 1.074 (1.057-1.091) | <0.001 |
| AST, U/L | -0.024 | 0.008 | 0.977 (0.962-0.992) | 0.002 |
| GGT, U/L | 0.108 | 0.010 | 1.114 (1.094-1.136) | <0.001 |
| ALP, U/L | 0.001 | 0.001 | 1.001 (1.000-1.003) | 0.020 |
| Serum urea, μmol/L | -0.074 | 0.039 | 0.929 (0.861-1.002) | 0.058 |
| Serum Cr, μmol/L | 0.003 | 0.005 | 1.003 (0.994-1.013) | 0.458 |
